# Primary Esophageal Diffuse Large B-Cell Lymphoma: A Comparative Review of 15 Cases

**DOI:** 10.1177/2324709618820887

**Published:** 2018-12-25

**Authors:** Faisal Inayat, Ahmed Munir, Ahsan Wahab, Fariha Younus, Fahad Zafar, Waqas Ullah

**Affiliations:** 1 Allama Iqbal Medical College, Lahore, Pakistan; 2 Services Institute of Medical Sciences, Lahore, Pakistan; 3 McLaren Regional Medical Center, Flint, MI, USA; 4 King Edward Medical University, Lahore, Pakistan; 5 Abington - Jefferson Health, Abington, PA, USA

**Keywords:** primary esophageal lymphoma, diffuse large B-cell lymphoma, incidence, diagnosis, management

## Abstract

Primary esophageal diffuse large B-cell lymphoma (DLBCL) is an extremely rare clinicopathologic entity. We report one case from our clinical experience and undertake a review of the previously published cases. A systematic literature search of the medical databases PubMed and Google Scholar was conducted. A total of 15 cases fulfilled the inclusion criteria. The data on patients’ characteristics, epidemiology, clinical features, HIV status, gross appearance of the lesion, esophageal location, treatment, and outcome were collected and analyzed. Primary esophageal DLBCL was more common among males, primarily in the fifth and sixth decades of life. Dysphagia was the most common initial clinical presentation. Tissue biopsy with immunohistochemistry was an indispensable diagnostic modality. The mainstay of treatment was chemotherapy with cyclophosphamide, vincristine, doxorubicin, and prednisone regimen, in addition to anti-CD20 antibody rituximab, with or without radiotherapy. This review serves to outline our current understanding of the epidemiology of and risk factors for primary esophageal DLBCL, the pathophysiology of this disorder, and currently available approaches to diagnosis and management.

## Introduction

Primary esophageal non-Hodgkin’s lymphoma is an uncommon gastrointestinal pathology that was first reported by Berman and colleagues in 1979.^1^ The incidence of this tumor is so infinitesimal that less than 30 cases have been described thus far.^2^ In one study, the incidence was only 0.2%, which was accounted by 3 cases of the total 1467 cases of primary gastrointestinal lymphoma.^3^ Esophageal involvement in lymphoma is predominantly due to the local invasion from gastric or mediastinal lesions and represents <1% of all cases of gastrointestinal lymphomas.^3^ Primary esophageal diffuse large B-cell lymphoma (DLBCL), a variant of non-Hodgkin’s lymphoma, is a rare tumor. It predominantly involves immunocompromised patients with HIV infection as a potential risk factor.^4^ Although the exact pathogenesis of primary esophageal DLBCL is unknown, the proposed mechanism implicates genetic aberrations in mature B cells. These cells normally differentiate in the bone marrow and migrate to the secondary lymphoid organs where antigen-dependent differentiation occurs. Several genetic alterations, occurring at any point in the pathway of differentiation, can contribute to their neoplastic transformation. These include downregulation of tumor suppressor genes like *p53, MYC*, and *BCL-2* or overactivation of proto-oncogenes like *BCL-6*.^4,5^

Primary esophageal DLBCL commonly presents with dysphagia and/or odynophagia.^6^ Initial detection of the lesion is carried out by radiological and endoscopic evaluations. Endoscopic ultrasound (EUS) with fine-needle aspiration (FNA) cytology helps to visualize the histopathologic architecture of the tumor. Moreover, immunohistochemistry (IHC) plays a key role in the differentiation from other benign and malignant lesions of the esophagus.^7^ With regard to the management, no specific guidelines exist. The chemotherapeutic regimen consisting of cyclophosphamide, doxorubicin, vincristine, and prednisone with rituximab (R-CHOP) has been used as the mainstay of treatment, which is occasionally followed by radiotherapy. The number of chemotherapeutic cycles depends on the severity and extent of the lesions. The patients with a limited disease usually receive 3 cycles, but those with an aggressive lymphoma undergo 6 to 8 cycles along with radiotherapy.^8^ In the present study, we chronicle a case of primary esophageal DLBCL where the diagnosis was established on the basis of pathognomonic histopathologic and IHC findings. Furthermore, we present a comparative review of this rare entity encompassing patient demographics, presenting signs and symptoms, investigative findings, treatment of individual cases, and clinical outcomes.

## Case Presentation

A 54-year-old female presented to our medical center with a 2-month history of progressive dysphagia and odynophagia. The patient experienced a sensation of solid foods “getting hung up” in her neck. However, she did not report choking or gagging. Before her current presentation, she underwent neck ultrasonography at the office of her primary care physician, which revealed a 6.1-cm complicated cyst. Considering it an infectious etiology, she was empirically treated with antibiotics, but her symptoms persisted. Her past medical history was significant for hypertension. She had no other complaints and there was no history of immunosuppressive disorders like HIV/AIDS, celiac disease, or inflammatory bowel disease. She denied nausea, vomiting, fever, chills, gastroesophageal reflux symptoms, or weight loss. Family history was negative for cancer. Physical examination was significant for a large, tender, and palpable neck mass.

### Investigations

**Figure 1.**
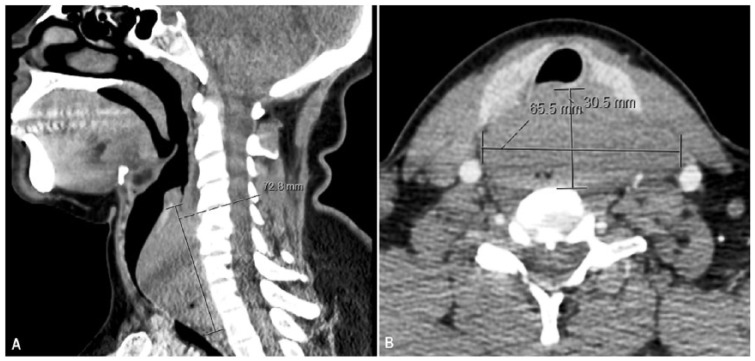
Computed tomography scan of the head and neck. (A) A 7.3 × 3.1 × 6.6-cm mass in the neck, located adjacent to the esophagus. (B) The lesion appeared to be closely apposed with the vertebral body without apparent compression.

**Figure 2.**
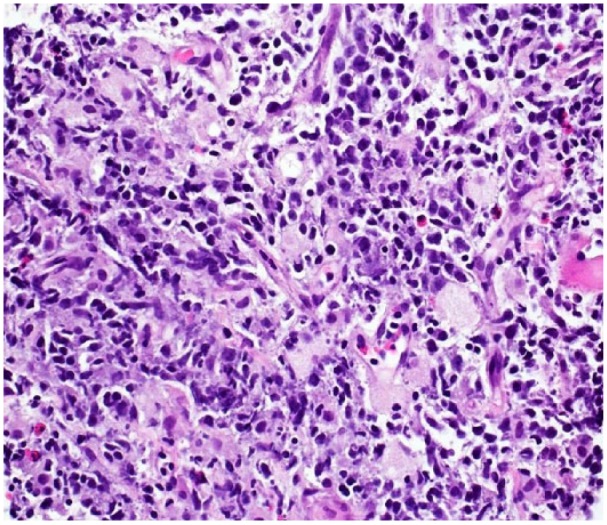
Photomicrograph of the biopsy specimen of the neck mass demonstrating large malignant lymphocytes with an amphophilic cytoplasm. The nuclei are round to ovoid with vesicular chromatin and variable number of nucleoli (hematoxylin-eosin; 400×).

**Figure 3.**
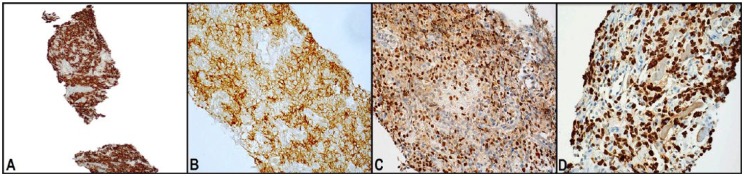
Immunohistochemical analysis of the biopsy of the neck mass. (A) Positive staining of malignant lymphocytes for the CD20 antibody (CD20; 100×). (B) Positive staining for the CD10 antibody (CD10; 400×). (C) Malignant lymphocytes were positive for the BCL6 antibody (BCL6; 400×). (D) The Ki-67 proliferative index was very high (up to 80%).

Laboratory studies for hematology, serum biochemistry, and urine analysis were within normal limits. Computed tomography (CT) scan of the head and neck showed a 7.3 × 3.1 × 6.6-cm mass in the neck, which was inseparable from the cervical esophagus (Figure 1A). It was located adjacent to the vertebral body without apparent bone compression (Figure 1B). Barium esophagogram showed an intact esophageal mucosa, with marked narrowing of the cervical part of the esophagus. Esophagogastroduodenoscopy confirmed the tumor-related esophageal luminal narrowing. EUS-guided FNA was performed, which came out inconclusive. Subsequently, the interventional radiology team performed an uneventful biopsy of the neck mass. Histopathologic analysis of the biopsy specimen showed relatively large malignant lymphocytes with a moderately abundant cytoplasm and round-to-ovoid nuclei having prominent nucleoli and occasional mitoses (Figure 2). Immunohistochemical examination yielded positive results for CD20 and CD10 antibodies, whereas staining was diffusely positive for BCL6 antibody (Figure 3A-C). On the basis of these pathologic findings, the patient was diagnosed with primary esophageal DLBCL. The lesion was classified as high grade due to a very high Ki-67 proliferative index (Figure 3D). A whole-body gallium scan was negative for other abnormalities. Bone marrow biopsy ruled out malignant changes. According to Ann Arbor staging, this was a stage IEA DLBCL with isolated esophageal involvement.

### Treatment

The patient was initiated on chemotherapy with R-CHOP regimen. She received a total of 4 chemotherapeutic cycles followed by radiotherapy.

### Outcome and Follow-up

She tolerated the treatment well without serious adverse events. The clinical response was good with resolution of dysphagia achieving complete remission in 3 months. During the 3-year follow-up period, the patient did not experience a recurrence of the lesion. She continues to do well to date.

## Discussion

Primary esophageal DLBCL is a variant of non-Hodgkin’s lymphoma with only a handful of cases reported thus far.^4^ We systematically searched the published medical literature in order to retrieve the available data. The articles were collected as of November 2018 using the medical databases PubMed (National Library of Medicine, Bethesda, MD) and Google Scholar. Different MeSH (Medical Subject Headings) terminologies, including “primary,” “diffuse large B-cell lymphoma,” and “esophagus,” were combined using the Boolean operators “AND” and “OR” with the terms “DLBCL,” “primary esophageal lymphoma,” and “treatment.” Furthermore, a few articles were retrieved through a manual search using the reference list of all accessible publications. The study inclusion criteria for the final comparative analysis comprised of the characteristics, 1) articles describing primary esophageal DLBCL, 2) availability of content in the full-text form, and 3) documentation of study findings in the English language.

A total of 57 articles consisting of but not limited to original articles, case series, and case reports were initially obtained using the above-mentioned search strategy. The titles and abstracts of all these articles were carefully reviewed for their relevance to our study. A total of 17 articles were first enlisted for re-review, while 40 studies were excluded as they were not related to primary esophageal DLBCL, were in a language other than English, and/or full-text versions were not available. After removing duplicate and redundant papers, 11 articles were included in the present study for the final review and analysis. A thorough reading of these articles yielded a total of 15 cases. The data of individual cases of primary esophageal DLBCL regarding epidemiology, clinical presentation, endoscopic findings, management, and outcome are summarized in Table 1. The data analysis demonstrated that the mean age of patients at the time of diagnosis was 56.1 years (range: 27-77 years). The tumor demonstrated male gender predominance (males, *n* = 11; females, *n* = 4). Clinical presentation was dominated by progressive dysphagia and unintentional weight loss. A few patients also reported constitutional symptoms. Although HIV infection has been deemed as a risk factor for gastrointestinal DLBCL, it was negative in 10 out of 15 cases included in this study. Therefore, it should also be suspected among HIV-negative individuals who present with symptoms and signs suggestive of esophageal malignancy. Among these patients, the distal esophageal involvement (*n* = 8) was more common compared with mid (*n* = 4) or upper segments (*n* = 3) of the esophagus.

**Table 1.** Demographic, Clinical, Endoscopic, HIV Status, Lesion Site, Treatment, and Outcome Data of Patients With Primary Esophageal Diffuse Large B-Cell Lymphoma.

Authors	Age/Gender/Country	Clinical Presentation	HIV Status	Gross Appearance	Esophageal Location	Treatment	Outcome
Bernal and del Junco^5^	40/male/USA	Dysphagia, weight loss	Positive	Ulcerated mass	Distal	Radiotherapy	NR
Moses et al^6^	55/male/Israel	Odynophagia, weight loss	Positive	Ulcer	Mid and distal	CHOP	Died during chemotherapy due to esophageal perforation
Weeratunge et al^7^	27/male/USA	Dysphagia, odynophagia, weight loss	Positive	Ulcer	Mid	Refused treatment	Died after 6 weeks
Weeratunge et al^7^	43/male/USA	Dysphagia, odynophagia, weight loss	Positive	Multiple ulcers	Diffuse	CHOP	Died after 4 weeks, during first cycle of chemotherapy
Chadha et al^8^	39/male/USA	Dysphagia, weight loss	Positive	Mass with nodular mucosa	Distal	Nd-YAG laser, m-BACOD	Died due to sepsis and pneumonia
Chadha et al^8^	76/female/USA	Dysphagia, weight loss	Negative	Mass with nodular mucosa	Upper	Radiotherapy	Died after 1 month due to airway obstruction
Chadha et al^8^	69/male/USA	Dysphagia, weight loss	Negative	Polypoid mass	Distal	R-CHOP	Complete remission
Sabljak et al^9^	42/female/Serbia	Dysphagia, weight loss	Negative	Esophageal obstruction with normal mucosa	Upper	Gastrostomy, R-CHOP 6 cycles, field irradiation therapy	Complete remission
Kalogeropoulos et al^10^	77/male/Greece	Epigastric pain, paroxysmal atrial fibrillation	Negative	Extensive rigid folds without erosions	Mid and lower	Combination chemotherapy 6 cycles	Complete remission
Ghimire et al^11^	41/male/China	Dysphagia	Negative	1.8 × 1.4-cm esophageal ulcer	Distal	Subtotal esophagectomy with gastric pull-up, R-CHOP 6 cycles, irradiation	Complete remission
Ghimire et al^11^	77/male/China	Dysphagia	Negative	Multiple solid, irregular, nodular lesions	Distal	Patient refused further investigations and treatment	Discharged on request
Mrad et al^12^	76/female/Lebanon	Dysphagia, weight loss	Negative	Tight stricture below cricopharyngeus	Upper	R-CVP 6 cycles	Complete remission
Castresana et al^13^	60/female/USA	Dysphagia, weight loss	Negative	Circumferential, ulcerated, multilobar esophageal mass at the GE junction	Distal	R-CHOP	Improvement after 2 cycles
Teerakanok et al^14^	60/male/USA	Dysphagia, cough, weight loss	Negative	Large fungating mass causing partial obstruction and TEF	Mid	PEG and esophageal tracheobronchial stent placement along with rituximab 1 cycle, CHOP 6 cycles	Good response in tumor, but persistent TEF with intermittent aspiration
Sugoor et al^15^	60/male/India	Dysphagia	Negative	An ulcero-proliferative friable mass with luminal narrowing	Distal	CEOP 6 cycles, radiotherapy	Complete remission
The present report	54/female/USA	Dysphagia, odynophagia	Negative	Esophageal lumen obstruction	Upper	R-CHOP 4 cycles, radiotherapy	Complete remission

Abbreviations: NR, not reported; Nd-YAG, neodymium-doped yttrium aluminum garnet; m-BACOD, methotrexate, bleomycin, doxorubicin, vincristine, cyclophosphamide, and dexamethasone; R-CHOP, rituximab-cyclophosphamide, doxorubicin, vincristine, prednisone; R-CVP, rituximab-cyclophosphamide, vincristine, prednisone; GE, gastroesophageal; TEF, tracheoesophageal fistula; PEG, percutaneous endoscopic gastrostomy; CEOP, cyclophosphamide, etoposide, vincristine, prednisone.

Primary esophageal DLBCL may pose a diagnostic dilemma due to the marked heterogeneity and nonspecificity observed in diagnostic investigations. In this review, barium swallow was frequently employed as an initial detection modality. CT neck and chest showed significant thickening of the esophageal wall causing luminal narrowing. Endoscopic evaluation showed a friable esophageal mass in most cases. Radiologically and endoscopically, these lesions may appear as ulcers, multiple submucosal nodules, strictures, enlarged folds, varicoid-like lesions, and rarely as aneurysmal dilatation.^16^ In these patients, EUS-guided FNA is a safe procedure and should be considered for initial detection of lymphoma as well as its differentiation from other esophageal disorders presenting with dysphagia.^17-19^ However, standard endoscopic biopsy, as in the present case, may have a low diagnostic yield. Interventional radiology may then be used to solve this diagnostic predicament.^20^ In selected patients, neck exploration or open biopsy may also be performed, if all other methods fail to make the correct diagnosis. In the current review, biopsy with IHC was used for the etiology establishment in all the patients. Pathologic examination showed lymphoid infiltration of the muscularis mucosa and adventitia, without infiltration of fat or sclerosis. IHC was mainly positive for tumor markers like CD10, CD20, CD45, CD 79a, PAX5, MUM1, and BCL6, and negative for BCL2, ALK, CD30, and cyclin D1. In most cases, the proliferation index (Ki-67 index) was more than 40% to 50%, which conferred a poor prognosis. 18F-FDG PET/CT was also performed in a few patients demonstrating an increased glucose uptake in the involved segment of the esophagus.

Due to rarity of the primary esophageal DLBCL, a standard treatment approach remains to be determined. The frequently used chemotherapy regimen, as in other subtypes of non-Hodgkin’s lymphoma, is R-CHOP.^21^ In the present review, most patients received 6 cycles that were occasionally followed by field irradiation for consolidation. Surgery in the form of subtotal esophagectomy was also performed in a few cases. Surgical intervention like open or minimally invasive esophagectomy can be undertaken in patients with early disease. However, in cases with late diagnosis, chemotherapy or irradiation is employed to shrink the size of the tumor before treating it with surgery. Similarly, surgery can also be performed in patients who develop complications or as a palliative treatment. Although radiotherapy is predominantly reserved for patients who are unable to tolerate chemotherapy, it may or may not be coupled with chemotherapy in cases with moderate-to-severe disease. Therefore, a case-by-case approach is applied in this regard. In terms of outcome analysis of the cases included in our review, 6 patients showed complete remission of the disease, whereas 2 showed significant improvement, 1 patient refused treatment, and exact outcome was not reported in 1 patient. Unfortunately, 5 patients died.

The exact trends of mortality and overall prognosis of primary esophageal DLBCL have not been deciphered. However, major prognostic factors include age at the time of diagnosis, functional status of the patient, presence of comorbidities (HIV infection), the presence of B symptoms, the nodal and extranodal extensions of the disease, serum lactate dehydrogenase levels, stage of the disease, and bone marrow involvement. Although the data are limited, the survival rates are observed to be ranging from 26% to 73%.^22^ In the present study, survival duration was not reported in a majority of cases (*n* = 9). In those patients where survival was documented (*n* = 5), it ranged from 1 week to 3 months and only 1 patient survived for over 3 years.

## Learning Points

- Primary esophageal DLBCL is a rare clinical entity with only a few cases described in the published medical literature.
- Endoscopic biopsy with histopathology and IHC is usually performed to identify these lesions. A deep tissue biopsy may also be required in cases where initial biopsies fail to yield a diagnosis.
- R-CHOP regimen is the mainstay of treatment in patients with primary esophageal DLBCL.
- The response to prompt chemotherapy with or without radiotherapy is considerably good, achieving complete remission in a majority of cases.
- Physicians should be vigilant for this tumor in not only immunocompromised but also in immunocompetent individuals presenting with consistent clinical features.
